# Exploring 6 years of colorectal cancer surgery in rural Italy: insights from 648 consecutive patients unveiling successes and challenges

**DOI:** 10.1007/s13304-024-01829-z

**Published:** 2024-04-17

**Authors:** Roberto Santoro, Marta Goglia, Manuela Brighi, Fabio Pio Curci, Pietro Maria Amodio, Domenico Giannotti, Angelo Goglia, Jacopo Mazzetti, Laura Antolino, Antonio Bovino, Costantino Zampaletta, Giovanni Battista Levi Sandri, Enzo Maria Ruggeri

**Affiliations:** 1 Unit of Oncologic and General Surgery, Belcolle District Hospital, Viterbo, Italy; 2 PhD in Training in Translational Medicine and Oncology, Department of Medical and Surgical Sciences and Translational Medicine, Faculty of Medicine and Psychology, Sapienza University of Rome, Rome, Italy; 3 Unit of Gastroenterology, Belcolle District Hospital, Viterbo, Italy; 4 Unit of General Surgery, Santa Scolastica District Hospital, Cassino, Italy; 5 Unit of Oncology, Belcolle District Hospital, Viterbo, Italy

**Keywords:** Colorectal cancer, Colorectal surgery, Colorectal cancer screening, Colorectal cancer mortality, Rural health, Rural areas

## Abstract

The multidisciplinary management of patients suffering from colorectal cancer (CRC) has significantly increased survival over the decades and surgery remains the only potentially curative option for it. However, despite the implementation of minimally invasive surgery and ERAS pathway, the overall morbidity and mortality remain quite high, especially in rural populations because of urban − rural disparities. The aim of the study is to analyze the characteristics and the surgical outcomes of a series of unselected CRC patients residing in two similar rural areas in Italy. A total of 648 consecutive patients of a median age of 73 years (IQR 64–81) was enrolled between 2017 and 2022 in a prospective database. Emergency admission (EA) was recorded in 221 patients (34.1%), and emergency surgery (ES) was required in 11.4% of the patients. Tumor resection and laparoscopic resection rates were 95.0% and 63.2%, respectively. The median length of stay was 8 days. The overall morbidity and mortality rates were 23.5% and 3.2%, respectively. EA was associated with increased median age (77.5 vs. 71 ys, *p* < 0.001), increased mean ASA Score (2.84 vs. 2.59; *p* = 0.002) and increased IV stage disease rate (25.3% vs. 11.5%, *p* < 0.001). EA was also associated with lower tumor resection rate (87.3% vs. 99.1%, *p* < 0.001), restorative resection rate (71.5 vs. 89.7%, *p* < 0.001), and laparoscopic resection rate (36.2 vs. 72.6%, *p* < 0.001). Increased mortality rates were associated with EA (7.2% vs. 1.2%, *p* < 0.001), ES (11.1% vs. 2.0%, *p* < 0.001) and age more than 80 years (5.8% vs. 1.9%, *p* < 0.001). In rural areas, high quality oncologic care can be delivered in CRC patients. However, the surgical outcomes are adversely affected by a still too high proportion of emergency presentation of elderly and frail patients that need additional intensive care supports beyond the surgical skill and alternative strategies for earlier detection of the disease.

## Introduction

Colorectal cancer (CRC) represents the third most common malignancy in the world in 2020 [1] and it was estimated to be the second most diagnosed cancer in Europe considering both sexes together [2]. In Italy, it ranks second for incidence and accounts for 11.6% of all cancers with more than 48.000 new cases estimated in 2022 and 21.700 deaths in 2021 [3]. The 5-year survival rate of patients who were diagnosed with CRC between 2005 and 2009 is 65%, significantly higher as compared to 52% reported in the 90's. The improvement of long-term survival can be attributed to both the multidisciplinary management of patients with advanced and metastatic disease [4] and on the efficacy of the screening program based on Fecal Occult Blood Test (FOBT), that provides diagnosis at an earlier stage [5, 6]. Surgery remains the only potentially curative option for it, and improvements in perioperative care and surgical techniques have brought additional advances. The minimally invasive approach and the enhanced recovery after surgery (ERAS) pathway have become common practice in high volume institutions reporting remarkable surgical results in selected population of patients in urban areas in elective setting approaching zero-mortality rates [7–10]. However, in a larger population scale including rural (less than 300 inhabitants/km^2^), urban (more than 1500 inhabitants/km^2^) and ‘intermediate regions’ the overall morbidity and mortality rates remain quite high approaching 4–5%, because of higher incidence of fragile and elderly patients, and emergency clinical presentation [11–15]. There are increasing evidence of the impact of rurality on access to state-of-the-art cancer prevention, diagnosis, and treatment services, as well as higher rates of risk factors [16]. For these reasons, the American Society of Clinical Oncology (ASCO) launched a rural cancer care initiative in 2018 to understand the factors contributing to cancer care disparities between rural and non-rural populations [17]. In 2021, according to EUROSTAT reports, 38.9% of the EU population was living in a city, with lower shares living in towns and suburbs (35.9%) and in rural areas (25.2%). In Italy, 27.6% of the population lives in rural areas with less than 150 inhabitants/km^2^.

The aim of this study is to analyze the characteristics and the surgical outcome of 648 CRC patients treated consecutively in two district hospitals serving similar large rural territories in Italy, with no selection bias.

## Patients and methods

All consecutive patients undergoing surgery for colorectal disease at the Department of Surgery of Santa Scolastica General Hospital in Cassino (Jan.2017–Feb.2020, named as Hospital #1), and Belcolle General Hospital in Viterbo (March 2020-Dec. 2022, named as Hospital #2), were enrolled in a prospective database, and those patients with a diagnosis of primary colon or rectal cancer (ICD-9-CM 153–154) are the object of this study. Patients’ characteristics, intraoperative data and surgical outcome were collected. The following factors were collected: age, sex, ASA score, hemoglobin level, stage of the disease, the type of hospitalization (emergency/elective) and the timing of surgery (emergency/elective), intraoperative findings, postoperative complications and in-hospital mortality, reoperations, length of hospital stay (LOS) and unplanned readmission within 30 days of discharge. Postoperative complications were classified according to Clavien-Dindo classification, as "procedure related" as anastomotic leak (AL), haemorrhage, intraabdominal abscess and wound infection or "general" as pulmonary and cardiac complications [18]. Primary outcomes included in-hospital morbidity and mortality, and secondary outcomes were laparoscopic resection rate, reoperation rate, LOS and 30-day unplanned readmission rate. In hospital mortality was defined as the death due to any cause during the hospitalization prior to discharge. Thirty-day readmission was defined as any unplanned, distinct hospitalization within 30 days after the discharge.

*Setting* *Hospital #1.* Santa Scolastica General Hospital in Cassino is situated in a large rural territory in the South part of the Lazio Region, with a population density of about 130 persons per km^2^, equidistant more than a hundred kilometers away from first level urban hospitals and can serve a population of more than 300 thousand people. The hospital has the amenities of second level hospital, including Gastroenterology and Internal Medicine Department, Intensive Care Unit and Department of Surgery with General Surgery, Gynecology and Orthopedics. The General Surgery Unit was dedicated to general surgery and emergency procedures with no experience in major oncologic surgery. A CRC surgery program was started in 2017 and joined a research group focused on the implementation of laparoscopic surgery and ERAS protocol [19]. A District interdisciplinary tumor board network was organized in collaboration with Oncology Unit, Radiotherapy Unit and Pathology Unit located in the other two hospital of the District.

*Hospital #2.* Belcolle General Hospital in Viterbo is situated in a similar large rural territory in the Northern part of the Lazio Region, with a population density of about 90 persons per km^2^ and serves a population of more than 300 thousand people, as well. The hospital has the amenities of a first-level hospital including Interventional Radiology and Surgical Endoscopy, Hemodynamics and Infectious Disease Unit and it was ranked among the high-volume institution for CRC surgery of the Region. A CRC screening program by fecal occult blood test (FOBT) and colonoscopy is running since 2014, and a multidisciplinary tumor board for the management of CRC was created in 2016, including diagnostic, therapeutic and ERAS pathways.

*Type of hospitalization and timing of surgery Emergency admission* (EA) was considered for those patients that were referred to the Surgical Unit from the Emergency Unit, and/or from the other medical Units of the hospital after diagnosis of CRC. Surgery was scheduled on elective basis unless emergency surgery (ES) was required in the event of non-deferability on the recommendation of the surgeon on call. Indication for emergency surgery was acute abdomen from perforation or worsening intestinal obstruction despite initial medical treatment by infusion therapy and nasogastric suction tube. Surgical procedure depended on the clinical conditions, age of the patient and on surgeon choice, according to Damage Control Surgery principles for patient safety [20].

*Elective admission* was planned for those patients that were referred to the Department clinics as outpatients by gastroenterologist or family doctors after endoscopic diagnosis of colorectal disease, because of the onset of light or mild symptoms or in case of screening detected disease.

*Preoperative workup* The preoperative assessment included colonoscopy with biopsy, thoraco-abdominal CT with contrast enhancement, tumor markers, preanesthetic evaluation and in selected case virtual colonoscopy and pelvic or liver MRI. Excluding patients operated on urgently, all patients were discussed before surgery in the CRC tumor board in order to define proper treatments according to ESMO Clinical Practice [21, 22]. Preoperative counseling was performed separately by the surgeon and the anesthesiologist. A cautious application of early recovery after surgery (ERAS) principles was applied with an increasing adherence during the study period thanks to continuous training and education of the surgical and nursing teams, as described in our previous study [9, 23].

*Surgical approach in elective procedure *Mechanical bowel preparation with oral antibiotic prophylaxis and thrombosis prophylaxis with low-molecular-weight heparin and elastic compression are routinely performed in all patients. Laparoscopic resection is the preferred technique of choice. After laparoscopic exploration of the abdominal cavity the procedures start with the primary ligation and section of the vascular pedicle followed by the posterior mobilization of the right or left colon. In case of right colectomy, the specimen is extracted through a small supraombelical midline incision and an extracorporeal double layer hand-sewn anastomosis is performed. In case of cancer involving the splenic flexure, resection is carried on in the same fashion used for right colectomy. Intracorporeal anastomosis was performed in selected cases and has become the technique of choice in 2023. In case of left colectomy or rectal resection, the specimen is extracted through a suprapubic transverse incision and a laparoscopic Knight-Griffen colorectal anastomosis is performed. A 18F silicon abdominal drainage was placed in regard of the anastomosis independent of the procedure in all patients. Diverting ileostomy is routinely performed in case of neoadjuvant treatment for extraperitoneal rectal cancer. In elderly patients or in case of bulky locally advanced cancers a standard open approach was preferred and a Hartmann colorectal resection with definitive stoma was usually performed in case of rectal cancer. Locoregional epidural analgesia has become the technique of choice in fragile patients.

*Postoperative treatment* ASA III, or more, and/or elderly patients received intensive care for at least 12 h in Intensive Care Unit after surgery. Nasogastric tube was removed at the end of surgery. Oral feeding with liquids was started on postoperative day (POD) 3 for the first year, and POD 2 afterword, and patients were discharged when able to satisfy the daily needs for mobility and nutrition. Postoperative infection criteria were hyperleukocytosis, combined with body temperature higher than 38.5 centigrade, positive biologic fluid cultures, such as blood, abdominal fluid, sputum. Thoraco-abdominal CT scan with intravenous and per os contrast enhancement was performed in all patients with infectious criteria to detect any sign of anastomotic leak, abdominal collection or pneumonia.

### Statistical analysis

Statistical analyses were performed with IBM SPSS Statistics for Windows (Ver. 26.0, NY: IBM Corporation). All data were expressed as mean ± standard deviation (SD) or median (interquartile range; IQR), otherwise specified. Normality was assessed by the inspection of frequency histograms. The unconditional logistic regression model was used for calculating the odds ratio (OR) with the estimation of 95% confidence interval (CI). Comparison of variables was performed with the Chi-square test and the Student's *t* test for categorical and continuous variables, respectively. Nonparametric analysis was performed with Mann − Whitney *U* test. All statistical tests were two-sided, a ‘‘*p*’’ value ≤ 0.05 was considered to indicate statistical significance.

## Results

**Fig. 1:**
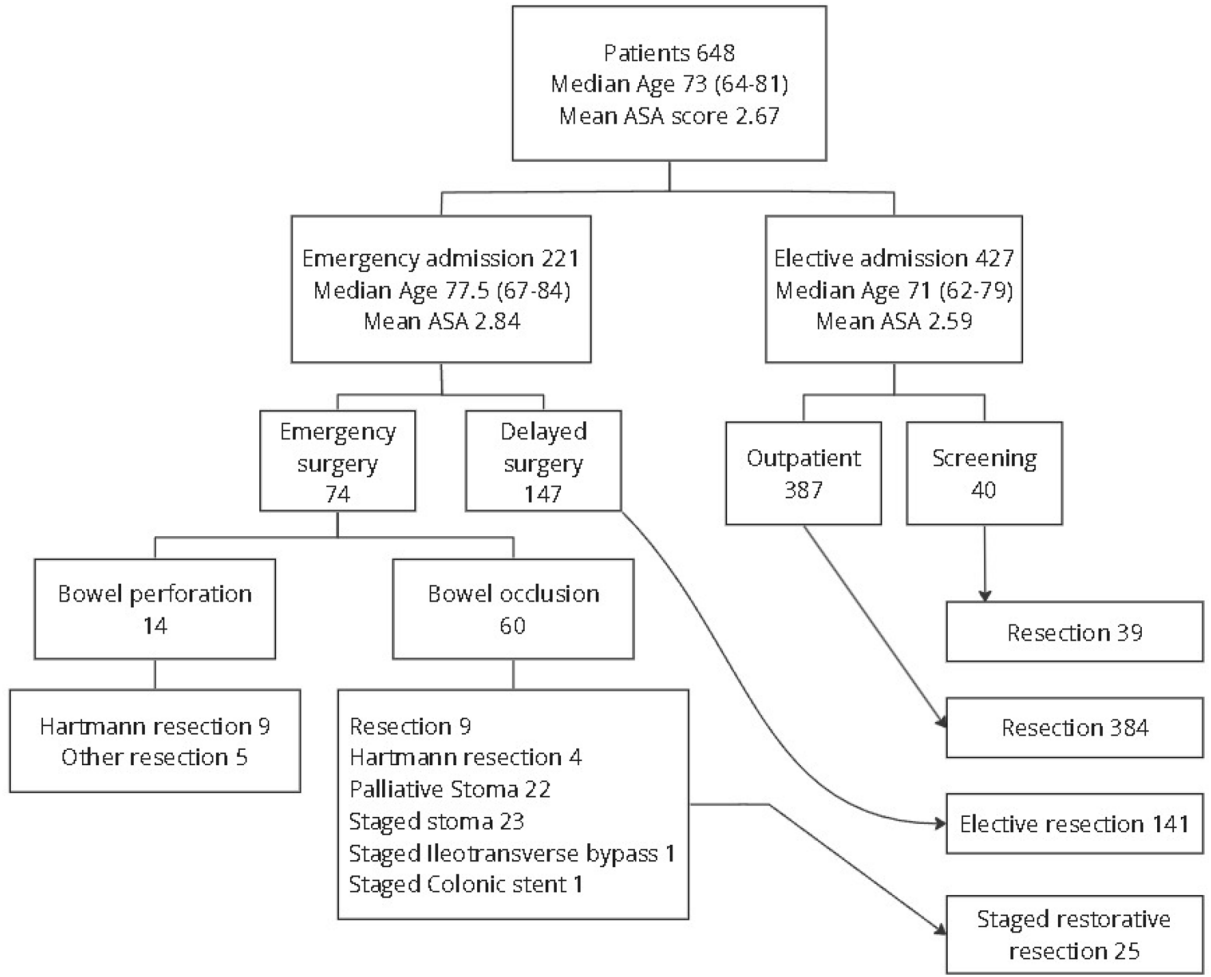
Flowchart of patient admission pathways and operative treatment

A consecutive series of 648 patients of a median age of 73 years (range 18–96) suffering from CRC were admitted at the Department of General Surgery with no selection bias, and the flowchart of the entire series is summarized in Fig. 1. Twenty-eight patients were younger than 50 years (4.3%). Emergency admission (EA) because of acute symptoms was recorded in 221 patients (34.1%). There were 354 male and 295 women (Table 1). Mean ASA score was 2.67 ± 0.72 and mean Hb level was 11.99 ± 2.18. The incidence of stage IV disease at diagnosis was 16.2%. Only 6.2% of the CRC diagnosis were detected by CRC screening program. The tumor was in the rectum in 211 patients (32.6%). Hospital #2 had an higher volume of procedures correlated with a higher number of elective admission (70% vs. 61.6%; *p* < 0.02), including younger and healthier patients recruited from a more effective screening program (1.9% vs. 10.2%; *p* < 0.001).

**Table 1:** - Patients and disease characteristics

	Nb. (%)	Hospital #1	Hospital #2	*p* value
Nb. Patients	648	315	333	
Hospital volume (pts/months)	9	8.3	9.7	
Nb. Emergency admission (%)	221 (34.1)	121 (38.4)	100 (30.0)	0.024
Female Gender (%)	295 (45.5)	141 (44.8)	154 (46.2%)	0.705
Age (median)	73 (IQR 64–81)	74 (IQR 65–81)	73 (IQR 63.5–73)	0.593
ASA score (mean)	2.67 ± 0.72	2.72 ± 0.77	2.62 ± 0.65	0.054
Hb (g/dL)	11.99 ± 2.18	11.72 ± 2.17	12.23 ± 2.16	0.001
Screening detected CRC (%)	40 (6.2)	6 (1.9)	34 (10.2)	0.000
Stage IV disease (%)	105 (16.2)	51 (16.2)	54 (16.2)	0.993
Tumor site: rectum (%)	211 (32.6)	94 (29.8)	117 (35.1%)	0.151

*ASA* American Society of Anaesthesiologists, *Hb* haemoglobin, *CRC* colorectal cancer

A total of 673 procedure were performed in 648 patients since a staged resection was carried on in 25 patients (Table 2). Tumor resection was carried on in 616 patients (95%). Right hemicolectomy or ileocecal resection were performed in 222 patients (34.2%), whereas resection of the transverse colon or left angle in 40 patients (6.3%). A left sided restorative resection was performed in 183 patients (29.7%), and resection of extraperitoneal rectal cancer requiring low colorectal anastomosis with temporary loop ileostomy was performed in 59 patients (9.6%). Hartmann procedure and Miles abdominal − perineal resection with definitive ostomy were performed in 60 (9.7%) and 14 (2.3%) patients, respectively. Subtotal colectomy was performed in 6 patients (0.9%). Multiple resections were performed in 15 patients. Segmental atypical resection and transanal minimally invasive surgery for large adenomas were carried out in 9 and 8 patients, respectively. Resection without anastomosis and VAC therapy was performed in one patient with severe septic shock from peritonitis. Non-resective procedures were performed as primary operation in 57 patients (8.8%), being palliative in 32 of them (4.6%). Complete laparoscopic resection was performed in 386 patients (62.7%), and conversion rate was 6.2%.

**Table 2:** Type of operation (673 procedures in 648 patients)

	*N* (%)
Tumour resection	616 (95.0)
Ileocecal resection	5 (0.8)
Right colectomy	217 (35.2)
Transverse colon resection	16 (2.6)
Splenic flexure resection	23 (3.7)
Left colectomy	85 (13.8)
Sigmoidectomy	48 (7.8)
Anterior rectal resection	50 (8.1)
Anterior rectal resection with ileostomy	59 (9.6)
Hartmann resection	60 (9.7)
Abdomino perineal resection	14 (2.3)
Subtotal colectomy	6 (0.9)
Multiple resections	15 (2.4)
Segmental \resections	9 (1.4)
TAMIS	8 (1.3)
Damage control resection + VAC	1 (0.1)
Non resective procedure	57 (8.8%)
Endoscopic stenting (followed by restorative resection)	1
Ileo-trasverse bypass (followed by restorative resection in one patient)	3 (0.4)
Staged diverting stoma (followed by restorative resection in all patients)	23 (3.4)
Palliative diverting stoma	28 (4.3)
Diagnostic laparoscopy	2 (0.3)
Laparoscopic resection	386 (63.2)
Conversion rate	24 (6.2)

*TAMIS* Transanal Minimally Invasive Surgery, *VAC* Vacuum Assisted Abdominal Closure

Overall morbidity (CD ≥ 1) and reintervention rates were 23.5% and 3.8%, respectively (Table 3). Cardiopulmonary and urinary complications and paralytic ileus were the most frequent problems after surgery, and 25 patients underwent reoperation for surgical complications, such as bleeding, anastomotic leak (AL), or occlusion. Particularly, after tumour resection, primary anastomosis was performed in 543 patients (88.1%), and the incidence of anastomotic leak (AL) was 3.1% (17 patients). Eleven of these patients required reoperation and 4 of them died because of sepsis. Postoperative mortality was correlated to the consequences of other surgical complications in two more elderly patients because of intraperitoneal and digestive bleedings. Overall mortality was 3.2% (21 patients), and in the remaining 15 patients the unfavorable outcome was due to complication of medical nature including the failure of preexisting cardiopulmonary or renal disease in fragile patients or the septic consequences of peritonitis in those patients that required emergency Hartmann procedure for perforation. In this subgroup of patients, the mortality rate was as high as 33%, as shown in Table 4. In case of bowel occlusion, postoperative mortality was 5%, while no postoperative mortality was recorded after staged surgery. In 427 elective procedures, mortality rate was 1.2% (Table 5), being 0% among those 40 patients belonging to the screening group.

**Table 3:** Postoperative outcomes in 648 patients

	*N* (%)
Patients without complications	496 (76.5)
Patients with post-operative complications	152 (23.5)
Clavien-Dindo Grade	
I	57 (8.8)
II	50 (7.7)
IIIa/IIIb	17 (2.6)
IV	8 (1.2)
Surgical complications	
Anastomotic leak/anastomosis	17/543 (3.1)
Abdominal abscess	15 (2.3)
Bleeding	31 (4.6)
Paralytic ileus	26 (4.0)
Urinary complication	28 (4.3)
Wound infection	13 (2.0)
Occlusion	3 (0.4)
General complications	
Cardio-pulmunary	34 (5.2)
Renalfailure	2 (0.3)
Mortality	21 (3.2)
Re-operation	25 (3.8)
Lenght of Stay (median)	8 (IQR 7–11)
(mean)	9.67 ± 5.66
Readmission rate	8 (1.2)

**Table 4:** Emergency surgery

	*N* (%)	Postoperative death
Patients	74 (11.4)	
Age (median)	79 (IQR 63.5–86)	
Tumor resections	27 (36.5)	
Perforation	14 (20.8)	5 (33%)
Tumor resection	14 (100%)	
Hartmann resection	9	4
DCS (resection + VAC)	1	1
Right colectomy (1 ileostomy)	2	
Left colectomy	2	
Bowel occlusion	60	3 (5%)
Tumor resection	13 (22%)	
Right colectomy	7	1
Left colectomy	2	
Hartmann resection	4	
Non resective procedure	47 (78%)	
Palliative diverting stoma	22	2
Temporary diverting stoma (staged surgery)	23	
Ileotransverse bypass (staged surgery)	1	
Endoscopic stenting (staged surgery)	1	
Morbidity (CD > 3)	11 (15.3)	
Mortality		8 (10.8)

*DCS* damage control surgery, *VAC* Vacuum Assisted Abdominal Closure, *CD* Clavien-Dindo Grade

**Table 5:** Comparison between elective and emergency admission patients

	Total	Emergency admission	Elective admission	OR	CI (95%)	*p*-value
Patients	648	221 (34.1)	427 (65.9)			
Age	73 (IQR 64–81)	77.50 (IQR 67–84)	71 (IQR 62–79)			0.000
Mean ASA score	2.67 ± 0.72	2.84 ± 0.72	2.59 ± 0.70		– 0.368 to – 0.131	0.002
Stage IV	105 (16.2)	56 (25.3)	49 (11.5)	2.618	1.712–4.004	0.000
Hb (g/dL)	11.99 ± 2.18	11.21 ± 2.05	12.37 ± 2.16		0.83028–1.53915	0.000
Rectal Cancer	211 (32.6)	70 (31.7)	141 (33)	0.940	0.664–1.331	0.729
Tumour resection	616 (95.1)	193 (87.3)	423 (99.1)	0.065	0.023–0.188	0.000
Restorative resection	541 (83.5)	158 (71.5)	383 (89.7)	0.288	0.188–0.442	0.000
Laparoscopic resection	390 (60.2)	80 (36.2)	310 (72.6)	0.224	0.159–0.317	0.000
Staged Surgery	25 (3.9)	25 (11.3)	0 (0)			0.000
Complications (CD ≥ 3)	46 (7.1)	26 (11.8)	20 (4.7)	2.713	1.4781–4.9809	0.0013
Mortality	21 (3.1)	16 (7.2)	5 (1.2)	6.587	2.380–18.231	0.000
LOS median (days)	8 (IQR 7–11)	9 (IQR 7–12)	8 (IQR 7–10)			0.470
Mean (days)	9.67 ± 5.66	9.9 ± 6.6	9.57 ± 5.09			

*OR* odds ratio, *ASA* American Society of Anaesthesiologists, *Hb* hemoglobin, *CD* Clavien-Dindo Grade, *LOS* Length of Stay

Median LOS of the entire series was 8 days, and it decreased from 9 days in the first two years to 7 days in the last two years. However, time to readiness for discharge (TRD) do not coincide with LOS, especially in those fragile or elderly patients that require long familiar and social support to recover and regain autonomy in basic life daily activities.

Thirty-day readmission rate was 1.2% (cholecystitis, urinary retention, occlusion, ileocolic anastomosis phlegmon and retroperitoneal abscess without AL). Only two patients required reoperation after discharge because of early dehiscence of aponeurotic plane, and a direct abdominal wall closure was required, and late AL that required salvage Hartmann procedure.

In Table 5, the univariate analysis shows that EA was associated with increased median age (77.5 vs. 71 years), mean ASA Score (2.84 vs. 2.59) and IV stage disease rate (25.2 vs. 11.9%), and lower mean hemoglobin (Hb) level (11.21 vs. 12.37 mg/dL). EA was also associated with lower tumor resection (87.3% vs. 99.1%), restorative resection (71.5 vs. 89.7%), and laparoscopic resection (36.2% vs. 71.7%) rates, as well as higher mortality rate (7.2% vs. 1.2%). Increased mortality rates were associated with ES (11.1% vs. 2.0%) and age more than 80 years (6.3% vs. 2%). The association of EA with age more than 80 years showed a significantly increased mortality rate compared to that of younger patients operated on after elective admission (10% vs. 0.6%).

In Table 6, all 648 patients were grouped according to the type of admission, timing of surgery and preoperative diagnostic pathway. As expected, the detected cancer patients coming from the screening program were significantly younger with earlier stage disease and the best surgical outcome with a median LOS of 7 days and no postoperative mortality. All variables worsen linearly towards the left columns, except the subgroup of patients that underwent a staged resection.

**Table 6:** Patients characteristics and surgical outcomes stratified according to the population

	Over all	Emergency admission			Elective admission	
		Emergency surgery	Staged surgery	Delayed Surgery	Outpatients	Screening
Patients	648	49 (7.6)	25 (3.9)	147 (22.7)	387 (59.6)	40 (6.2)
Age median	73 (IQR 64–81)	79 (IQR 65–87)	71 (IQR 62.25–79.25)	77 (IQR 67–84)	73 (IQR 63–80)	65 (IQR 60–69)
ASA	2.67 ± 0.72	3.08 ± 0.731	2.6 ± 0.645	2.77 ± 0.708	2.61 ± 0.716	2.45 ± 0.54
Stage IV disease	105 (16.2)	19 (38.8)	5 (20)	32 (21.6)	46 (11.9)	3 (7.5)
Tumour resections	616 (95)	27 (55.1)	25 (100)	141 (95.9)	384 (99.2)	39 (97.5)
Restorative resections	541 (87.8)	13 (26.5)	24 (96)	121 (82.3)	347 (89.7)	36 (90)
Laparoscopic resections	390 (60.2)	3 (6.1)	14 (56)	63 (42.7)	273 (71.1)	37 (92.5)
Complications (CD > 3)	46 (7.1)	11 (22.5)	3 (12.0)	12 (8.2)	19 (4.9)	1 (2.5)
Mortality	21 (3.2)	8 (16.3)	0 (0)	8 (5.4)	5 (1.3)	0 (0)
LOS median (days)	8 (IQR 7–11)	7 (IQR 6–13.25)	7 (IQR 7–16)	9 (IQR 7–12)	8 (IQR 7–10)	7 (IQR 6–8)

## Discussion

The strength of this study is typical rural territorial context served by the hospitals and the absence of selection bias. The most interesting evidence of the study is that the main outcomes are consistent with those of other larger Western countries and Italian population-based studies [11–15, 24–28] in which more than 30% of the patient population had an emergency hospitalization, and almost 50% of the patients population was 75 years or older. Particularly, the evaluation of Italian hospital data on whole Italian population in 2015 showed that, in a total of 27.019 patients operated on for CRC in 604 hospitals, the overall 30-day mortality was 4.1% [15], and in another large study on more than 350.000 patients living in Veneto and operated on between 2005 and 2014, in-hospital mortality was 2.5% and LOS was 13 days [12]. However, the present study shows that, despite the improvements in perioperative care and surgical techniques that provided excellent results in elective hospitalizations approaching almost zero-mortality rate, the overall morbidity and mortality rates remain high since complications occur almost exclusively in the large subgroup of patients that undergo surgery after emergency hospitalization as shown in Table 6.

Several rurality-associated factors contribute to rural–urban disparities in cancer care and outcomes. Rural populations tend to be older, have lower educational attainment, and lower median household income compared with nonrural residents and in CRC patients, a higher emergency hospitalization rate and more advanced stage of the disease have been shown compared to patients residing in urban areas [16]. In our experience, the emergency admission rate was 34.1% and it increased with patients' age, being 46% in ultraoctagenarian patients. In this group of patients are concentrated the most fragile ones that are burdened with the worst surgical outcomes, because of age, comorbidities, and the acute impairing of general conditions, as shown in Table 5. Emergency surgery was required in 11.4% of the patients and mortality was mainly correlated to the devastating septic consequences of peritonitis from perforation at admission. However, despite these failures, the postoperative mortality rate of 10.8% after emergency surgery was very satisfactory due to the cautious surgical strategy we adopted in case of bowel occlusion. Performing the "old" decompressing cecostomy in 45 patients (or stenting in one case) as first-line emergency operation, both for palliative management or as bridge to curative surgery, turned out to be a safe and cost-effective procedure. The staged resection was possible in 23 of these patients and it turned out to be an advantageous strategy, compared to Hartmann procedure or resection with risky primary anastomosis, since it allowed to perform a laparoscopic elective restorative resection in half of the patients safely in our series, taking advantage of the protection of the stoma with no mortality [29–31]. Endoscopic stenting was also shown to be very effective in case of bowel occlusion and should be proposed as primary option, however, in our experience we observed that it remains a demanding and difficult procedure to organize in rural hospital for reasons of different nature, especially in an emergency setting. Cecostomy was the definitive treatment in 22 patients unfit for surgery because of the association with metastatic disease, advanced age, and poor performance status. Thus, our study suggests that whatever the context, rural or urban, decompressive cecostomy, even though it represents one of the oldest procedures in the history of surgery, it allowed to perform the most modern procedures in the second step. During our experience we have been extremely impressed by the sudden improvement of such critical patients provided by this simple procedure performed in locoregional epidural anesthesia in most of our patients. Indeed, it allowed the use of minimally invasive approach in the second surgical step and the “staged surgery" can be recommended as the standard approach in case of obstructive CRC especially for those surgeons who face such situations in Spoke Units. Such "staged resection" appears to be worthy on the long term too, over the traditional sequence of difficult operations following standard Hartmann procedure, such as the challenging reversal of Hartmann's procedure and the frequent need of incisional hernia repairs that have high morbidity rates [32].

After emergency admission, delaying surgery in elective setting was possible in two thirds of patients. During hospitalization the diagnostic protocol was completed, and the patient's conditions could be improved in order to face the most appropriate surgical intervention in best possible health conditions and, hopefully, reduce the incidence of postoperative complications. This group of patients consist mainly of a geriatric population, with impaired functional reserve and lower chance to recover in case of surgical complication. However, older age is not a contraindication for surgery, since elderly patients form a varied group ranging from individuals with very good health status to those with limited life expectancy due to advanced cancer disease or poor performance status. Frailty status has been proposed as a superior measure for risk-stratifying patients, and the Comprehensive Geriatric Assessment can be helpful in the decision making by predicting complication in patients undergoing elective CRC surgery [33]. In a such compromised population of frail patients, a recent randomized clinical trial on the effect of multimodal pre-habilitation involving exercise, nutritional and psychological intervention failed to demonstrate a benefit on 30-day postoperative complications undergoing resection of CRC [34], and frailty is also a predictor of poor outcome, even after uneventful operation [35]. When considering all these critical issues, tumor resection with definitive stoma creation turns out to be the safest surgical option in such frail patients suffering from non-metastatic rectal cancer, accounting for 23% of the procedure in elderly patients in our experience, as well as palliative stoma in case of stage IV disease. The overall mortality rate of 6.3% in 191 ultraoctagenarian patients can be considered very satisfactory, and unfortunately, an "inevitable" price to pay in CRC surgery. In our more recent experience, frail patients underwent "awake surgery" with favorable outcomes and in our opinion locoregional epidural anesthesia can become a standard indication in the future not only for frail patients [36, 37]. It is evident that the association of these two independent risk factors (i.e. emergency admission and age > 80 years) is correlated to a significant increase of postoperative mortality up to 10% and this subgroup of patients needs a tailored organization and more effective multidisciplinary supports, representing "the dark side of the moon" in CRC surgery.

Our experience spanning over 6 years in two district hospitals with similar surrounding territory shows that the number of admissions from the emergency room is similar as well, and depends essentially on the number of inhabitants in the surrounding area. In fact, in case of emergency clinical situation patients require hospitalization in the nearest hospitals independently from the quality of cares. Thus, low-volume rural centers treat paradoxically the worst group of our CRC population, as shown in the left columns of Table 6. In the second center, we registered an increase of elective hospitalization for CRC compared to the historical data before 2020. Indeed, patients living in the surrounding territory and coming from other regions, especially those patients recruited from screening programs, accounted for up to 40% of the elective admissions. In our opinion, the increase of elective hospitalization might be due to the significant implementation of the minimally invasive surgical approach that represents today the standard of care even in patients’ expectations [38].

Among rural–urban cancer care disparities, the availability of oncology infrastructure, gastroenterologist and colorectal surgeon, have been advocated. In fact, high volume hospitals are typically concentrated in metropolitan areas, and a strict correlation between hospital (or surgeon) volume and surgical outcome has been reported in several studies [12, 13, 27]. In our country, a minimal volume of 50 cases per year was shown to represent the cutoff line to keep mortality under 5%. Unfortunately, only 27.6% of the hospitals were reported to have such minimal volume of procedures in 2015, and more than 10.000 patients were operated on in low volume hospitals, that paradoxically deal more frequently with this type of patients for reasons of proximity, especially for those serving vast rural territories, as already mentioned [15]. The shift of colon and rectal cancer patients to high-volume surgeons or high-volume urban hospitals has been suggested [11, 12, 27], since high volume hospital are undoubtedly prepared to deal with postoperative complications such as anastomotic leak, that remains the main concern for the surgeon, and sepsis [39, 40]. However, in a such widespread oncological disease over the country, the shift of colon and rectal cancer patients to urban high-volume hospitals might have disadvantages for the patients’ safety and comfort in population residing in rural areas and will have an unjustifiable negative impact on health economics. We agree that implementing the activity in medium and low volume district hospitals up to a hundred procedure per year by hiring a high-volume surgeon represents a more profitable alternative to shifting the patients to referral centers, as shown in our previous study [23]. Public health strategies based on this proposal would bring great benefit to the entire population especially those residing in rural areas, that cannot cope with the inconveniences and costs of referral to tertiary centers because of social and familial problems, including the challenges with transportation. In addition, in our country, the outcomes of CRC surgery have become indicators of the effectiveness and quality of care of the hospital and shifting the patients also represents a detriment to the hospital’s clinical and administrative performance [41–43]. However, referral to tertiary center, or high-volume surgeon, should be considered for patients with extraperitoneal rectal cancer that remains a challenging surgical procedure. From this point of view, the development of the oncologic regional networks is highly recommended and will properly define the patients’ pathways. In our experience, in the second district hospital, a local oncologic network is running from 2020 and those patients who require emergency hospitalization in the three Spoke hospitals of the area are managed in collaboration with referral Hub high volume center of the area depending on patient’s clinical condition. According to internal protocol, those patients that require emergency surgery are treated in the Spoke hospitals and then referred to the Hub in order to guarantee the best treatment in any kind of presentation. Moreover, in this new innovative network, the surgical teams of the Spoke hospitals as well move from the Spokes to the Hub to take care of the patients, integrating their selves with the Hub surgical team [44].

So far, hospital accreditation programs have been developed on the basis of surgical volume and the oncologic network mainly implies the referral of patients to the metropolitan high volume centers without any kind of cooperation between surgical teams. Our innovative model of cooperation and integration between the surgical teams residing in hub and spoke centers of the same territory represents a valid alternative especially in rural areas able to increase the volumes and improve the quality of cares [45].

When diagnosis of CRC is made on an outpatient basis, elective surgery can be scheduled, and the patients can be included in the ERAS pathway. In a such selected population of younger and healthier patients the surgical outcome becomes remarkable, approaching zero mortality with high laparoscopic resection rates [7–10]. In our series, the median age of the group of patient undergoing resection after elective hospitalization was significantly younger compared to that of the patients operated on after emergency admission (71 vs. 77 years), however, median age is still significantly higher compared to that reported in other Italian urban single center series (67 years) [8, 10], where the incidence of patients aged less than 50 years can reach 10% [46]. In this larger group of patients, the laparoscopic resection rate reached 71.7%, and the postoperative mortality and LOS were 1.2% and 8 days, respectively (Tab. 5). As expected, the association of elective hospitalization with age younger than 80 years showed a further reduction in the mortality rate to 0.6%, similarly to urban referral centers [7, 9, 10]. Developing and implementing the laparoscopic technique and the ERAS program requires a hard work involving the surgical team and nursing staff, as well as all other professionals during the preoperative, perioperative and postoperative phases of the procedure, especially in a rural context where a perioperative care modification can be perceived as a complete revolution [47]. In our experience, the minimally invasive technique has been rapidly accepted because of the evident results in term decreased postoperative pain and shortened hospitalization and recovery times, whereas the ERAS pathway needs more time because of factors of different nature depending on the different items. In this context, we maintained the routine use of preoperative bowel preparation and abdominal drainage, and beside this practice, a high ERAS adherence was feasible in this selected group of patients, that unfortunately, represented less than 50% of our patient’s population. A cautious and tailored application of the ERAS principles rapidly provided excellent results without affecting postoperative outcome, as demonstrated by the reduction of the median LOS during the study period. However, the time to readiness for discharge (TRD) do not coincide with LOS, because of factors of nonclinical nature, such as social and family issues and the type of sanitary system organization, and were shown to be progressively longer with increasing age [10, 48]. This is particularly true in a rural context, as shown in this study, where the mean age is significantly higher and social criticism might be increased because of distances and family component migration compared urban areas.

Among the numerous barriers to the delivery of high-quality oncologic care for rural populations, the lower density of gastroenterologists and general surgeons was shown to adversely impact colorectal cancer screening services and colorectal cancer outcomes [16]. Effective strategies of health literacy intervention on repeat CRC screening of rural community clinics are recommended and will improve the surgical outcome and survival in working-age and active population on the long term [5, 6, 49, 50]. However, frail and elderly patients living isolated in the countryside still risk remaining forgotten, and will continue to present at emergency with advanced stage CRC and poor performance status. Thus, urgent identification strategies, that place more responsibility on family members or family doctors, are badly needed to detected CRC at an earlier stage in these patients and improve the surgical outcome in the short term.

In conclusion, after the analysis of a 6-year-real-life experience in the management of CRC in rural populations in Italy, we learned that hospitals serving large rural territories can deliver high quality oncologic cares. However, we also learned that morbidity and mortality rates remain high due to a still too high incidence of emergency presentation of frail patients. All supportive cares, beyond the surgical skill, are needed to improve the outcome in this large subgroup of patients that can be considered the "dark side of the moon" in CRC surgery. The development of a local oncologic network with a fair collaboration among surgical teams is mandatory especially in rural context.
